# Supplementary material for: Fate of Surgical Patients with Small Nonfunctioning Pancreatic Neuroendocrine Tumors: An International Study Using Multi-Institutional Registries
Source: Cancers (Basel). 2022 Feb 18;14(4):1038. doi: 10.3390/cancers14041038 (PMC8870171; doi:10.3390/cancers14041038)
Supplement: Supplementary file 1 [file cancers-14-01038-s001.zip › cancers-1541684-supplementary.pdf]

Supplementary Materials: Fate of Surgical Patients with Small Nonfunctioning Pancreatic Neuroendocrine Tumors: An International Study Using Multi-Institutional Registries

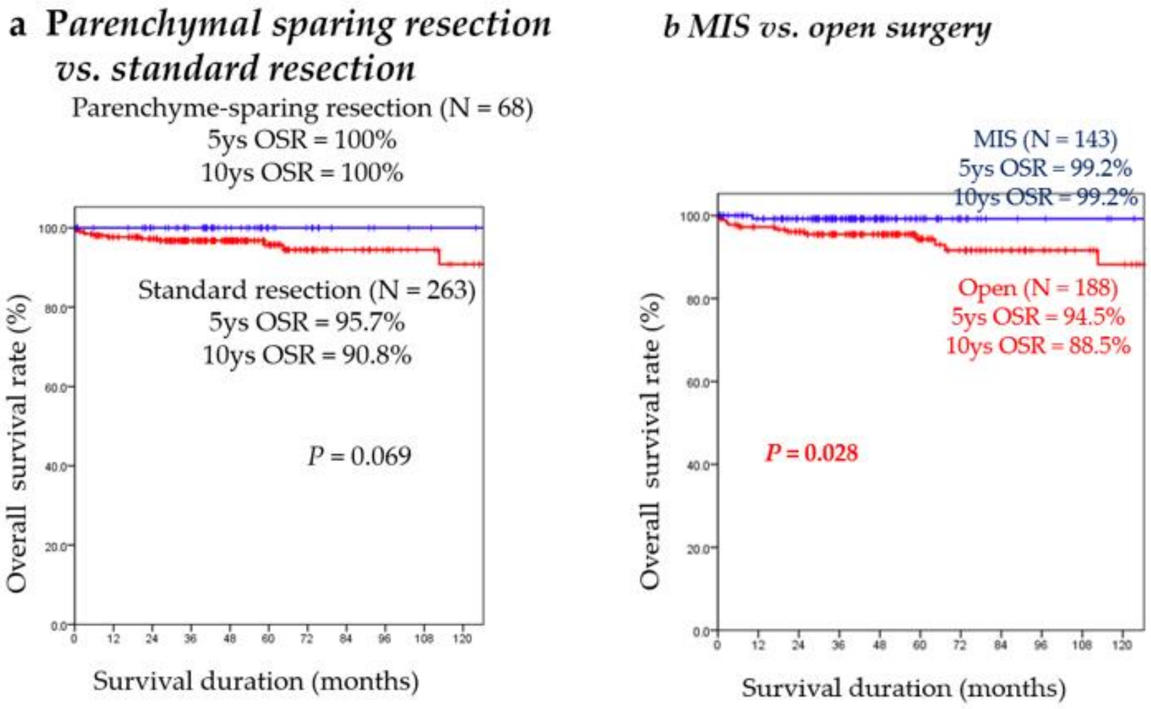

Figure S1. The overall survival on Korean patients for type of surgery.

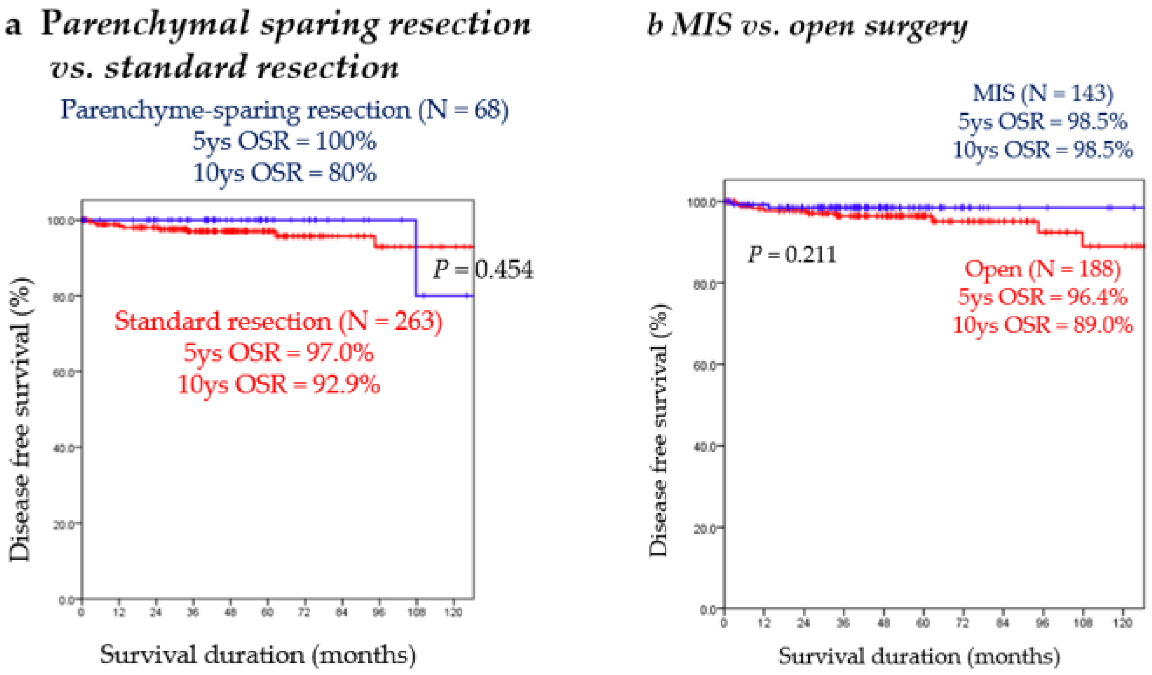

Figure S2: The recurrence-free survival on Korean patients for type of surgery.

Table S1. Postoperative complications following parenchymal-sparing resection and standard resection.

Complication (*N*, %)	Parenchymal-sparing resection (*N*= 67)	Standard resection (*N*= 262)	*p- Value*
Major complications	4 (6.0)	24 (9.2)	0.404
Cardiovascular	0	0	
Pulmonary	0	6 (2.3)	0.354
Cerebrovascular	0	1(0.4)	1.000
Renal failure	0	0	
POPF	4 (6.0)	21 (8.0)	0.573
Delayed gastric emptying	1 (1.5)	10 (3.8)	0.473
Hemorrhage	0	8 (3.1)	0.367
Intra-abdominal abscess	0	2 (0.8)	1.000
Wound problem	0	3 (1.1)	1.000

POPF: postoperative pancreatic fistula

Table S2. Postoperative complications following minimally invasive and open resection

Complication (*N*, %)	Minimally invasive resection (*N*= 141)	Open resection (*N*= 188)	*p-* Value
Major complications	12 (8.5)	16 (8.5)	1.000
Cardiovascular	0	0	1.000
Pulmonary	6 (4.3)	0	0.006
Cerebrovascular	0	1 (0.5)	1.000
Renal failure	0	0	1.000
POPF	11 (7.8)	14 (7.4)	0.904
Delayed gastric emptying	3 (2.1)	8 (4.3)	0.363
Hemorrhage	4 (2.8)	4 (2.1)	0.730
Intra-abdominal abscess	0	2 (1.0)	0.508
Wound problem	0	3 (1.5)	0.263

POPF: postoperative pancreatic fistula

Table S3. Distribution of risk factors for survival

Variables (*N*, %)	WHO grade				Tumor size			Ki-67 index			
	**G1**	**G2**	**G3**	***P***	**≤1.5cm**	**> 1.5cm**	***P***	**<3**	**3-20**	**>20**	***P***
Age (years)				0.017			0.952				0.010
≤65	293 (80.5)	92 (87.6)	6 (54.6)		272 (81.4)	121 (81.2)		315 (81.2)	63 (86.3)	4 (44.4)	
>65	71 (19.5)	13 (12.4)	5 (45.5)		62 (18.6)	28 (18.8)		73 (18.8)	10 (13.7)	5 (55.6)	
Tumor size				0.103							0.066
≤1.5cm	260 (71.4)	65 (61.9)	6 (54.6)					277 (71.4)	45 (61.6)	4 (44.4)	
> 1.5cm	104 (28.6)	40 (38.1)	5 (45.5)					111 (28.6)	28 (38.4)	5 (55.6)	
WHO grade							0.103				<0.001
G1					260 (78.6)	104 (69.8)		364 (93.8)	0 (0)	0 (0)	
G2					65 (19.6)	40 (26.9)		23 (5.9)	72 (98.6)	0 (0)	
G3					6 (1.8)	5 (3.4)		1 (0.3)	1 (1.4)	9 (100)	
Ki-67 index							0.066				
<3	364 (100)	23 (24.2)	1 (9.1)	<0.001	277 (85)	111 (77.1)					
3-20	0 (0)	72 (75.8)	1 (9.1)		45 (13.8)	28 (19.4)					
>20	0 (0)	0 (0)	9 (81.8)		4 (1.2)	5 (3.5)					
Mitosis				<.001			0.026				<.001
<2	364 (100)	35 (50.7)	2 (28.6)		285 (93.1)	116 (86.6)		369 (95.1)	32 (71.1)	0 (0)	
≥2	0 (0)	34 (49.3)	5 (71.4)		21 (6.9)	18 (13.4)		19 (4.9)	13 (28.9)	5 (100)	
Nodal metastasis				<.001			0.069				<.001
No	332 (96.8)	82 (83.7)	6 (54.6)		290 (94.5)	132 (89.8)		353 (96.5)	55 (82.1)	4 (44.4)	
Yes	11 (3.2)	16 (16.3)	5 (45.5)		17 (5.5)	15 (10.2)		13 (3.6)	12 (17.9)	5 (55.6)	
Vascular invasion				<.001			0.001				<.001
No	326 (92.9)	80 (80.8)	7 (63.6)		298 (92.8)	118 (82.5)		346 (92.3)	57 (85.1)	5 (55.6)	
Yes	25 (7.1)	19 (19.2)	4 (36.4)		23 (7.2)	25 (17.5)		29 (7.7)	10 (14.9)	4 (44.4)	
Perineural invasion				<.001			0.231				<.001
No	323 (92.6)	84 (86.6)	5 (50)		291 (91.5)	124 (87.9)		347 (93)	54 (83.1)	3 (37.5)	
Yes	26 (7.5)	13 (13.4)	5 (50)		27 (8.5)	17 (12.1)		26 (7)	11 (16.9)	5 (62.5)	
